# Retrograde nerve growth factor signaling modulates tooth mechanical hyperalgesia induced by orthodontic tooth movement via acid-sensing ion channel 3

**DOI:** 10.1038/s41368-021-00124-6

**Published:** 2021-06-04

**Authors:** Meiya Gao, Xinyu Yan, Yanzhu Lu, Linghuan Ren, Shizhen Zhang, Xiaoqi Zhang, Qianyun Kuang, Lu Liu, Jing Zhou, Yan Wang, Wenli Lai, Hu Long

**Affiliations:** 1 State Key Laboratory of Oral Diseases & National Clinical Research Center for Oral Diseases & Department of Orthodontics, West China Hospital of Stomatology, Sichuan University, Chengdu, China; 2 Department of Orthodontics, Shanxi Provincial People’s Hospital, Taiyuan, China

**Keywords:** Ion channels, Single-molecule biophysics

## Abstract

Orthodontic tooth movement elicits alveolar bone remodeling and orofacial pain that is manifested by tooth mechanical hyperalgesia. Nerve growth factor (NGF) is upregulated in periodontium and may modulate tooth mechanical hyperalgesia. The objectives were to examine the role of NGF in tooth mechanical hyperalgesia and to elucidate the underlying mechanisms. Tooth mechanical hyperalgesia was induced by ligating closed coil springs between incisors and molars in Sprague–Dawley rats. Retrograde labeling was performed by periodontal administration of fluor-conjugated NGF and the detection of fluorescence in trigeminal ganglia (TG). Lentivirus vectors carrying NGF shRNA were employed to knockdown the expression of NGF in TG. The administration of agonists, antagonists, and virus vectors into TG and periodontium was conducted. Tooth mechanical hyperalgesia was examined through the threshold of biting withdrawal. Our results revealed that tooth movement elicited tooth mechanical hyperalgesia that could be alleviated by NGF neutralizing antibody and that NGF was upregulated in periodontium (mainly in periodontal fibroblasts) and TG. Retrograde labeling revealed that periodontal NGF was retrogradely transported to TG after day 1. Acid-sensing ion channel 3 (ASIC3) and NGF were co-expressed in trigeminal neurons and the percentage of co-expression was significantly higher following tooth movement. The administration of NGF and NGF neutralizing antibody into TG could upregulate and downregulate the expression of ASIC3 in TG, respectively. NGF aggravated tooth mechanical hyperalgesia that could be alleviated by ASIC3 antagonist (APETx2). Moreover, NGF neutralizing antibody mitigated tooth mechanical hyperalgesia that could be recapitulated by ASIC3 agonist (GMQ). NGF-based gene therapy abolished tooth mechanical hyperalgesia and downregulated ASIC3 expression. Taken together, in response to force stimuli, periodontal fibroblasts upregulated the expressions of NGF that was retrogradely transported to TG, where NGF elicited tooth mechanical hyperalgesia through upregulating ASIC3. NGF-based gene therapy is a viable method in alleviating tooth-movement-induced mechanical hyperalgesia.

## Introduction

Orofacial pain, with a prevalence of 16% among general population,^1^ is a constellation of painful conditions in the orofacial regions and comprises trigeminal neuralgia, headaches, temporomandibular joint disorders, and dental pain.^2,3^ Of particular, orofacial pain induced by orthodontic tooth movement is manifested by mechanical hyperalgesia in affected teeth.^4,5^ It has been well-documented that patients’ masticatory functions are negatively influenced by orofacial pain induced by tooth movement.^6^ However, to date, no truly effective pain-relief treatment is available for tooth mechanical hyperalgesia induced by tooth movement.^4^ This is partly attributed to poor understanding of its underlying mechanisms, justifying further in-depth mechanistic elucidation.

Nerve growth factor (NGF), an important protein in the process of neurogenesis and neuron growth,^7^ plays an important role in pain modulation.^8^ It has been revealed that the expression of NGF in periodontal tissues was upregulated in response to tooth movement.^9^ Our previous study revealed that periodontal administration of NGF could cause tooth mechanical hyperalgesia and that periodontal administration of NGF neutralizing antibody mitigated tooth mechanical hyperalgesia,^10^ suggesting that NGF participates in the modulation of tooth mechanical hyperalgesia. It has been revealed that acid-sensing ion channel 3 (ASIC3) is expressed in periodontal Ruffini body that is a mechanical sensory structure.^11^ Our previous studies found that ASIC3 participates in the modulation of orofacial pain induced by tooth movement in both periodontal tissues and trigeminal ganglia.^12,13^ Moreover, the expressions of ASIC3 in sensory neurons could be regulated by NGF.^14^ All these findings suggest that ASIC3 could be a downstream of NGF in modulating tooth mechanical hyperalgesia. However, whether NGF modulates tooth mechanical hyperalgesia through ASIC3 is still poorly understood. Therefore, in this study, we aimed to examine the mechanisms whereby NGF modulates tooth mechanical hyperalgesia.

## Results

### The upregulation of NGF in periodontium after tooth movement

**Fig. 1:**
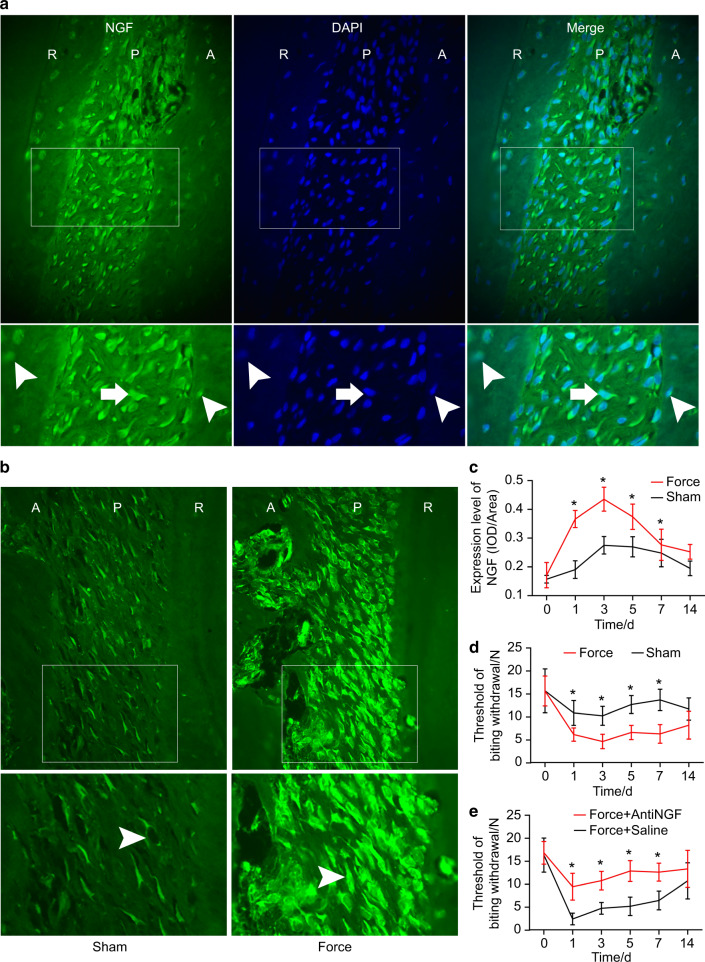
Orthodontic tooth movement elicited elevated expressions of NGF and induced mechanical hyperalgesia. **a** Expression patterns of NGF in periodontium on day 1 after orthodontic tooth movement. The lower panel shows a higher magnification of the box-selected region. NGF was expressed in periodontal fibroblasts (arrows), cementocytes (arrowheads in root area), and osteocytes (arrowheads in alveolar bone). **b** The comparison of NGF expressions between the force and sham group on day 3. The lower panel shows a higher magnification of the box-selected region. NGF immunostaining was more intense in the force group than in the sham group (arrowheads). **c** The expression levels of NGF were significantly higher on days 1, 3, 5, and 7 in the force group than in the sham group. **d** Orthodontic tooth movement resulted in mechanical hyperalgesia. The threshold of bite withdrawal was significantly lower in the force group than in the sham group on days 1, 3, 5, and 7. **e** Neutralizing NGF antibody relieved tooth mechanical hyperalgesia. The threshold of biting withdrawal was significantly higher in the Force+anti-NGF group than in the Force + Saline group on days 1, 3, 5, and 7. A, alveolar bone; P, periodontium; R, root; *P < 0.05

In periodontal tissues, as displayed in Fig. 1a, NGF was expressed in cementocytes, osteocytes, and fibroblasts in periodontium, with fibroblasts being the major cells positive for NGF. Following tooth movement, the expression levels of NGF started to increase on day 1, peaked on day 3, gradually decreased after day 5, and almost returned to the baseline level on days 7 and 14 (Fig. 1b, c).

### Tooth movement elicited NGF-dependent tooth mechanical hyperalgesia

As shown in Fig. 1d, the threshold of biting withdrawal was significantly lower in the force group than in the sham group on days 1, 3, 5, and 7 following tooth movement, indicating that tooth mechanical hyperalgesia was induced by tooth movement. Moreover, the threshold of biting withdrawal was significantly higher in the force + anti-NGF group than in the force + saline group on days 1, 3, 5, and 7, indicating that NGF was implicated in tooth mechanical hyperalgesia (Fig. 1e).

### The upregulation of NGF in TG after tooth movement

**Fig. 2:**
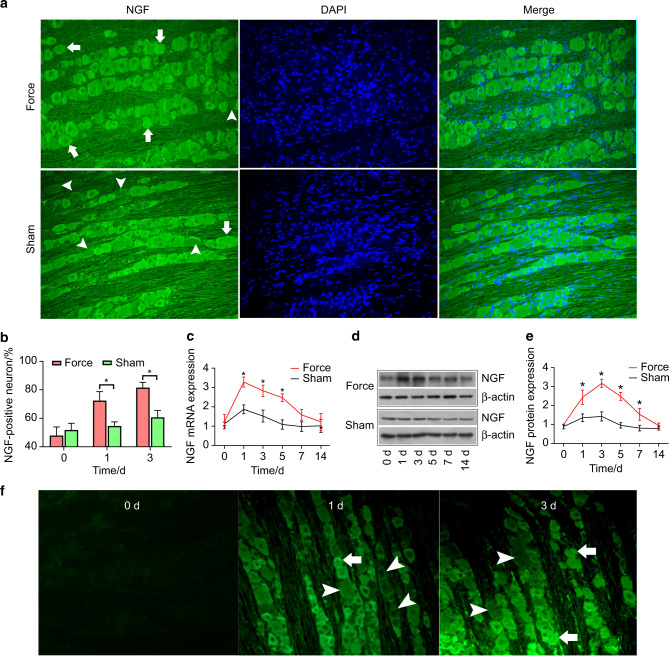
Orthodontic tooth movement elicited upregulation of NGF in trigeminal ganglia (TG) and induced retrograde transport of NGF. **a** The expression patterns of NGF in TG (magnification: ×200). NGF was more intensely immunostained in the force group than in the sham group on day 3. Note that more NGF-positive neurons and fewer NGF-negative neurons were in the force group. **b** The percentage of NGF-positive neurons was significantly higher on day 1 (*P* = 0.01) and day 3 (*P* < 0.01) in the force group than in the sham group. **c** The real-time PCR revealed that the expression level of NGF mRNA in TG was significantly higher in the force group than in the sham group on days 1, 3, and 5. **d**, **e** Western blotting detected that the expression level of NGF protein was significantly higher in the force group than in the sham group on days 1, 3, 5, and 7. **f** Fluor 488-conjugated NGF was injected in periodontium and started to be detected in TG on day 1. More immunofluorescence-conjugated NGF was detected on day 3. Arrows, NGF-positive neurons; arrowheads, NGF-negative neurons; *P < 0.05

As shown in Fig. 2a–e, real-time PCR, western blotting, and fluorescence quantification all revealed that the expression levels of NGF were elevated in TG following tooth movement. The percentage of NGF-positive neurons was significantly higher on day 1 (72.4% ± 6.3% vs. 54.6% ± 2.9%) and day 3 (81.6% ± 3.7% vs. 60.7% ± 4.8%) in the force group than in the sham group. Moreover, both real-time PCR and western blotting revealed that the expression levels of NGF were significantly higher in the force group than in the sham group.

### Retrograde transport of NGF

Our results revealed that, following tooth movement, the fluorescence signals of NGF were firstly detected in TG on day 1 following the periodontal injection of the fluoro-conjugated NGF (Fig. 2f).

### NGF modulated tooth mechanical hyperalgesia through ASIC3

**Fig. 3:**
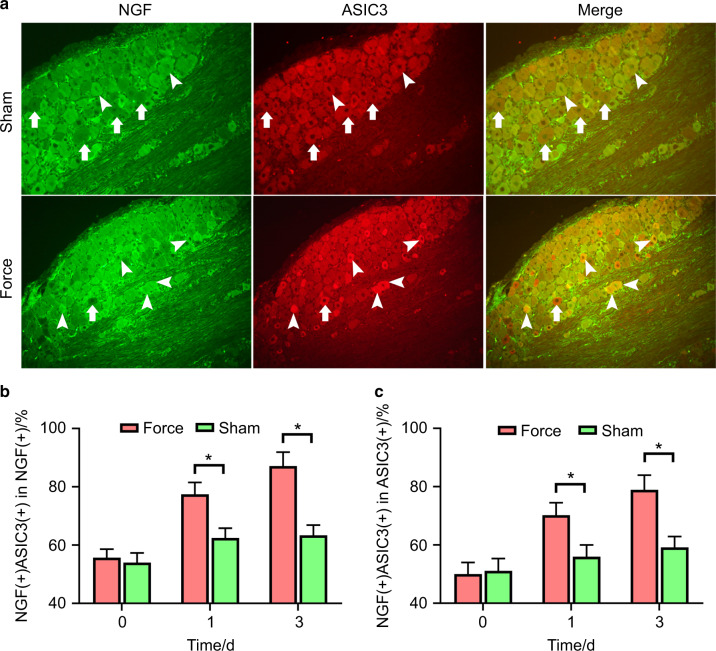
Orthodontic tooth movement promoted co-expression of NGF and ASIC3. **a** Co-expression of NGF and ASIC3 in trigeminal neurons on day 3 following tooth movement. More neurons were co-expressed with NGF and ASIC3 in the force group as compared to the sham group. **b** The percentage of co-positive neurons among NGF-positive neurons was significantly higher in the force group than in the sham group on days 1 and 3 (*P* < 0.001). **c** The percentage of co-positive neurons among ASIC3-positive neurons was significantly higher in the force group than in the sham group on days 1 (*P* = 0.004) and 3 (*P* = 0.0003). Arrows, non-co-expression neurons; arrowheads, co-expression neurons; *P < 0.05

As depicted in Fig. 3a–c, we found that the percentage of NGF + ASIC3-positive neurons among NGF-positive neurons was significantly higher on day 1 (77.44% ± 3.34% vs. 62.48% ± 2.75%) and day 3 (87.11% ± 3.92% vs. 63.36% ± 2.85%) in the force group than in the sham group. Moreover, the percentage of NGF + ASIC3-positive neurons among ASIC3-positive neurons was significantly higher on day 1 (70.20% ± 3.55% vs. 55.96% ± 3.30%) and day 3 (78.93% ± 4.11% vs. 59.19% ± 3.05%) in the force group than in the sham group.

**Fig. 4:**
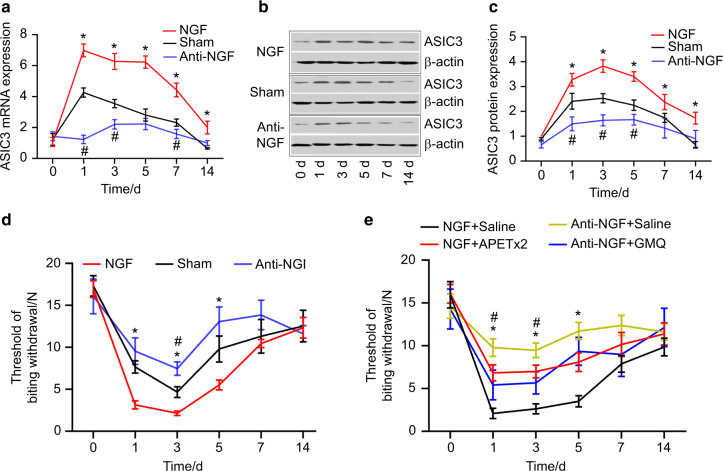
NGF modulated tooth mechanical hyperalgesia through ASIC3. **a** Real-time PCR revealed that the expression level of ASIC3 mRNA was significantly higher in the NGF group than in the sham group on days 1, 3, 5, 7, and 14 and was significantly lower in the anti-NGF group than in the sham group on days 1, 3, and 7. **b**, **c** Western blotting revealed that the expression level of ASIC3 was significantly higher in the NGF on days 1, 3, 5, 7, and 14. ASIC3 expression level was significantly lower in the anti-NGF group than in the sham group on days 1, 3, and 5. **d** NGF aggravated while NGF neutralizing antibody alleviated tooth mechanical hyperalgesia. The threshold of biting withdrawal was significantly lower in the NGF group than in the sham group on days 1, 3, and 5 and significantly higher in the anti-NGF group than in the sham group on day 3. **e** APETx2 (ASIC3 antagonist) alleviated NGF-induced mechanical hyperalgesia and GMQ (ASIC3 agonist) exacerbated anti-NGF-alleviated mechanical hyperalgesia. The threshold of biting withdrawal was significantly higher in the NGF + APETx2 group than in the NGF + Saline group on days 1, 3, and 5. The threshold of biting withdrawal was and significantly lower in the anti-NGF + GMQ group than in the anti-NGF + saline group on days 1 and 3. *,^#^*P* < 0.05

Both the PCR and western blotting revealed that NGF and NGF neutralizing antibody were able to upregulate and downregulate the expression of ASIC3 in TG, respectively (Fig. 4a–c).

The administration of NGF into TG could exacerbate tooth mechanical hyperalgesia while NGF neutralizing antibody (anti-NGF) was able to attenuate tooth mechanical hyperalgesia (Fig. 4d). Moreover, as depicted in Fig. 4e, we found that ASIC3 antagonist (APETx2) could attenuate NGF-aggravated mechanical hyperalgesia and that ASIC3 agonist was able to exacerbate mechanical hyperalgesia that was attenuated by anti-NGF neutralizing antibody.

### NGF-based gene therapy alleviated mechanical hyperalgesia via downregulating ASIC3

**Fig. 5:**
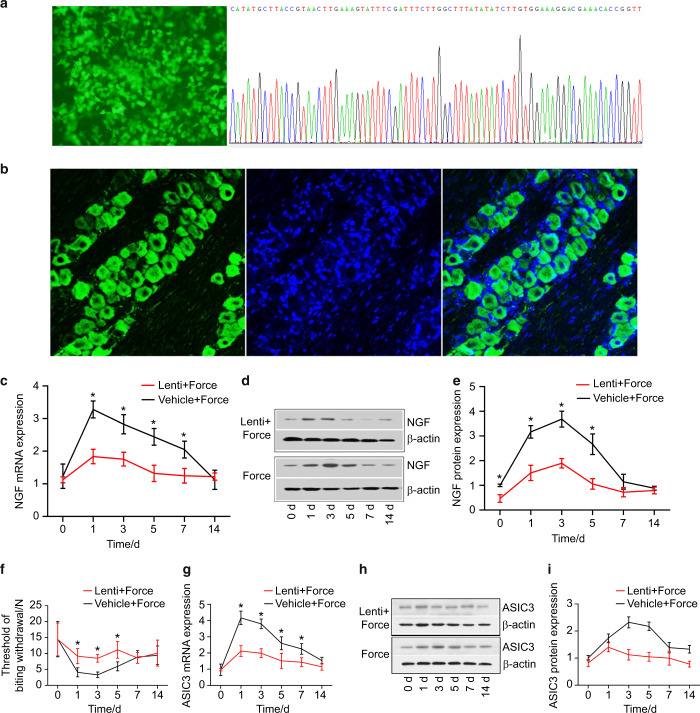
NGF-based gene therapy alleviated mechanical hyperalgesia via downregulating ASIC3. **a** The transduction of lentivirus vector into 293T cells was successful. The left figure shows the fluorescence field. The right figure reveals the confirmation of NGF shRNA sequence through DNA sequencing. **b** Lentivirus vector was transduced into trigeminal neurons in trigeminal ganglia (TG) 7 days following the administration of lentivirus vector into TG. The lentivirus vector carrying NGF shRNA was successful in silencing NGF in TG (**c** real-time PCR, **d**, **e** western blotting). **f** Lentivirus transduction alleviated mechanical hyperalgesia. The threshold of biting withdrawal was significantly higher in the lenti + force group than in the vehicle + force group on days 1, 3, and 5. NGF knockdown downregulated the expression of ASIC3 in TG (**g** real-time PCR; **h**, **i** western blotting). *P < 0.05

As displayed in Fig. 5a, the DNA sequencing confirmed the success of transduction into 293T cells and the NGF RNAi sequence in the lentivirus vector. Our results showed that green fluorescence was detected in TG 7 days following virus transduction, indicative of the success of virus transduction (Fig. 5b). We found that the expression level of NGF in TG was significantly lower in the lenti + force group than in the vehicle + force group on days 1, 3, and 5 following tooth movement (Fig. 5c–e). The threshold of biting withdrawal was significantly higher in the lenti + force group than in the vehicle + force group on days 1, 3, and 5 (Fig. 5f). Moreover, our results revealed that the expression level of ASIC3 was significantly lower in the lenti + force group than in the vehicle + force group (Fig. 5g–i).

## Discussion

Tooth movement in response to force stimuli involves a cascade of inflammatory reactions in periodontal tissues, including vascular changes, the recruitment of immune cells and the release of pro-inflammatory mediators.^4^ The resulting pro-inflammatory mediators, e.g., H^+^ and BMP-2, on one hand initiate local bone remodeling and on the other hand bond to nerve terminals to elicit painful sensations and mechanical hyperalgesia.^12,15,16^ Tooth mechanical hyperalgesia is the most prominent symptom associated with tooth movement, manifested with biting or chewing pain and masticatory disturbance.^3^ In our previous study, we invented the methods of using bite force for the evaluation of mechanical hyperalgesia induced by tooth movement and validated the viability and reliability of this method.^10^ Interestingly, we found that the threshold of biting withdrawal was slightly decreased in the sham group, which could be attributed to discomfort caused by the ligations of intraoral springs on teeth. Our preliminary study revealed that periodontal administration of NGF could reproduce tooth mechanical hyperalgesia and NGF neutralizing antibody was able to mitigate mechanical hyperalgesia induced by tooth movement.^10^ However, the underlying mechanisms in modulating tooth mechanical hyperalgesia were elusive. In this study, we conducted a thorough study on the mechanisms whereby NGF modulates tooth mechanical hyperalgesia.

NGF, a 130-kDa protein, was firstly found to be responsible for nerve growth and repair.^7,17^ In-depth investigations revealed that NGF played an important role in pain modulation.^18^ Local administration of NGF could elicit pain and neutralizing antibody against NGF could significantly alleviate pain,^8^ rendering neutralizing antibody treatment against NGF a good pain-relief option in clinical practice.^19^ Released by local injured tissues, NGF elicits profound inflammatory reactions that ultimately cause painful sensations.^20^ This is consistent with our results that the expression level of NGF in periodontal tissues was elevated following tooth movement and that periodontal administration of NGF could induce tooth mechanical hyperalgesia. The local mechanism of NGF production at injury remains elusive. It was accepted that two types of cells may be responsible for the local production of NGF: mast cells (in soft tissues) and osteoblasts (in skeletal bone).^21,22^ In contrast to soft tissues and skeletal tissues, periodontal tissue is a specialized tissue that could initiate more profound bone remodeling due to its abundant blood and nerve supply. Moreover, the predominant cells residing in periodontal tissues are fibroblasts (also designated as periodontal ligament cells) that play an important role in periodontal and bone remodeling in response to tooth movement.^23,24^ In our study, we found that the majority cells that expressed NGF in periodontal tissues were fibroblasts that play an important role in mechanotransduction of orthodontic force.^25^ The study by Tomlinson et al. revealed that, in response to mechanical loading, osteoblasts expressed and released NGF to sensory terminals that in turn release neuropeptides.^22^ The neuropeptides released from sensory terminals actively participate in alveolar bone remodeling.^26,27^ Of particular, CGRP is one of the most important neuropeptides that plays important roles in bone remodeling and periodontal sensation.^28–30^ Our previous study showed that periodontal administration of NGF could upregulate the expression of CGRP in periodontal tissues and administration of anti-NGF neutralizing antibody downregulate its expression.^10^ We suggest that, in response to orthodontic force, periodontal fibroblasts express and release NGF to periodontal sensory terminals, which in turn release CGRP to periodontal tissues to participate in periodontal remodeling and sensation. Furthermore, we previously found that the knockdown of ASIC3 in the trigeminal ganglia through RNA interference could downregulate the expression of CGRP.^13^ Consistently, it was reported that the activation of ASCI3 could promote the release of CGRP from trigeminal neurons.^31^ Conceivably, NGF-ASIC3-CGRP signaling pathway may exist and ASIC3 may be an intermediate molecule between NGF and CGRP. However, this notion should be validated with further studies.

Interestingly, it was suggested that local NGF were retrogradely transported to TG to elicit downstream events.^20^ In line with this study, we found that fluorescence signals were detected on days 1 and 3 following the periodontal administration of Fluor 488-conjugated NGF, indicating that periodontal NGF was retrogradely transported to TG and could reach TG within 1 day. Moreover, in our present study, we found that NGF expression was elevated in TG following tooth movement (Fig. 2). However, it is elusive whether the elevated expression of NGF is totally or partially contributed by periodontal NGF. The study by Kurata et al. shed light on this question and revealed that, following tooth movement, NGF mRNA could be detected in TG by in situ hybridization.^32^ This finding suggests that NGF could be de novo synthesized in TG following tooth movement, which is supported by our results that the peaks of NGF expressions were on day 3 for both TG and periodontal tissues. Thus, we suggest that the upregulated NGF in TG is partially contributed by periodontal NGF.

Our previous study revealed that ASIC3 participates in the pain modulation induced by tooth movement.^12^ ASIC3 is an ion channel that can be activated by proton and mechanical stimuli.^33,34^ In-depth research unraveled that ASIC3 was expressed in periodontal Ruffini body (a mechanosensory structure located in periodontal tissues),^11^ justifying a possible role of ASIC3 in the modulation of mechanical hyperalgesia. A large body of evidence shows that ASIC3 participates in the modulation of mechanical hyperalgesia.^35,36^ In our another study, we found that ASIC3 participated in the modulation of tooth mechanical hyperalgesia induced by tooth movement and that ASIC3-based gene therapy knocking down the expression of ASIC3 in TG was able to attenuate tooth mechanical hyperalgesia.^13^ Thus, in our present study, we examined whether ASIC3 was the downstream molecule of NGF responsible for tooth mechanical hyperalgesia. First, we found that NGF and ASIC3 were co-expressed in TG and that the percentage of their co-expression increased following tooth movement, indicative of their possible interaction effects. Then, our results revealed that intra-ganglionic administration of NGF could elevate the expression levels of ASIC3 while the administration of anti-NGF neutralizing antibody decreased ASIC3 expression, indicating that NGF was able to upregulate the expression of ASIC3 in TG. This finding was in accordance with the study by Mamet et al. where NGF was shown to upregulate the expression of ASIC3.^14^ Moreover, as a downstream factor, ASIC3 agonist was able to exacerbate mechanical hyperalgesia attenuated by anti-NGF neutralizing antibody. Further mechanistic study revealed that NGF upregulated the expression of ASIC3 through c-jun/p38MAPK pathway and that ASIC3 was crucial for the development of NGF-induced mechanical hyperalgesia.^37^ Consistent with their studies, we found that ASIC3 agonist recapitulated tooth mechanical hyperalgesia that was attenuated by periodontal administration of anti-NGF neutralizing antibody. Moreover, our results revealed that ASIC3 antagonist could attenuate tooth mechanical hyperalgesia that was aggravated by periodontal administration of NGF. Thus, we suggest that NGF modulates tooth mechanical hyperalgesia through upregulating ASIC3. Although NGF exerts its function through autocrine or paracrine mode in trigeminal ganglia,^32^ evaluation of the co-localization of TrkA (NGF receptor) and ASIC3 would be better for demonstrating the interaction between NGF and ASIC3. We did not assess the co-localization of TrkA and ASIC3 in this present study and this is a limitation of our study.

Last, we examined the effectiveness of NGF-based gene therapy in alleviating tooth mechanical hyperalgesia. The immunostaining revealed that the lentivirus vectors were successfully transduced into TG. The expression levels of NGF were downregulated in the lentivirus + force group, indicative of the success of knockdown of NGF in TG in vivo. Moreover, we found that the knockdown of NGF resulted in a downregulation of ASIC3 in TG, which further supports the aforementioned result that NGF regulated ASIC3 expression. NGF-based gene therapy alleviated tooth mechanical hyperalgesia induced by tooth movement.

## Conclusion

**Fig. 6:**
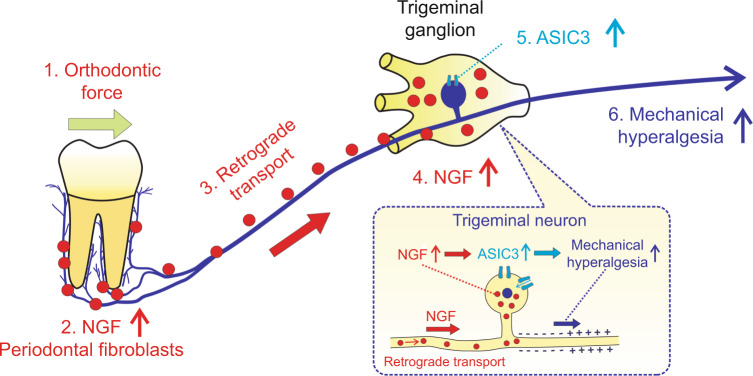
A schematic illustration depicting the mechanisms of tooth-movement-induced mechanical hyperalgesia. Once applied on teeth, orthodontic force stimulates the upregulation of NGF in periodontal fibroblasts; periodontal NGF was then retrogradely transported from periodontium to trigeminal ganglia where NGF elicits mechanical hyperalgesia through ASIC3

In conclusion, as displayed in Fig. 6, in response to mechanical stimuli, periodontal fibroblasts express NGF that can be retrogradely transported to TG. NGF in TG participates in the modulation of mechanical hyperalgesia through ASIC3. Gene therapy based on NGF is able to alleviate mechanical hyperalgesia.

## Materials and methods

### Animals and the induction of tooth movement

Male Sprague–Dawley rats (200–300 g) were obtained from the Animal Experimental Center of Sichuan University and housed in an air-conditioned room at 21 °C with a 12-h day–night cycle. Standard rat chow and water were provided ad libitum. This study was approved by the Ethical Committee of West China Hospital of Stomatology, Sichuan University (WCHSIRB-2017-052). This study adhered to the ARRIVE guidelines for conducting animal research.

Tooth movement was induced by ligating closed coil springs between incisors and molars. Specifically, following general anesthesia with pentobarbital sodium (50 mg·kg^−1^), rats were placed in supine positions and the closed coil springs were stretched and activated to deliver 40-g force. For sham group, closed coil springs were ligated but not activated (0-g force).

### Evaluating tooth mechanical hyperalgesia through threshold of biting withdrawal

Tooth mechanical hyperalgesia was evaluated through a bite-force measurement device according to our previous studies.^5,10^ Briefly, after being acclimated and trained to bite the biting sensors, rats were moved from a distance (10 cm) toward the bite sensor to elicit their biting on the biting sensor. The bite force was measured by using a bite-force measurement device (50.00 N ± 0.03 N; Nanjing Bio-inspired Intelligent Technology Co., Ltd., Nanjing, China). The bite force was measured for three trials and the maximal bite force was regarded as the threshold of biting withdrawal for that testing session and used for statistical analyses. The bite force was assessed averagely 4 h after drug administration when rats were fully awake.

### Retrograde fluorescence tracing

Fluorescence-labeled NGF with Alexa Fluor 488 was prepared by using Alexa Fluor® 488 Protein Labeling Kit (Thermo Fisher Scientific) according to manufacturer’s protocols. NGF conjugated with Alexa Fluor 488 (15 µL) was injected into periodontal tissues. Rats were sacrificed and trigeminal ganglia (TG) obtained for the examination of fluorescence.

### Administration of drugs

The administration of drugs into TG of rats was conducted according to our previous study.^38^ In brief, following general anesthesia with pentobarbital sodium (50 mg·kg^−1^), rats were placed in lateral recumbent positions and micro-injection needles were injected between tympanic bulla and the posterior border of mandibular ramus. The direction of the needle was mediocaudally and perpendicular to the long axis of the body. Needles were advanced by 9 mm to reach the TG, and drug suspension (10 µL) was injected over 1 min. For the administration of drugs into periodontal tissues, drug suspension (15 µL) was injected at three sites (5 µL for each), i.e., labial, palatal, and mesial (incisor) or distal (molar). Drugs were administered 4 h prior to the assessment of bite force on days 1, 3, 5, 7, and 14. Specifically, the concentrations of NGF, anti-NGF neutralizing antibody, APETx2, and GMQ were 0.4 µg·µL^−^^1^, 0.8 µg·µL^−^^1^, 0.1 µg·µL^−1^, and 0.05 µg·µL^−1^, respectively.

### Preparation of lentivirus vectors

After linearization, lentivirus vectors (ubiquitin promoter) encoding enhanced green fluorescence protein were recombined with the RNAi sequence (TTGGAGATAAGACCACAGCCA) of NGF. The recombinants were amplified through PCR, and the sequences were verified through DNA sequencing. Then, vector packaging and harvesting were performed by transfection of 293T cells, followed by titer quantification through quantitative PCR. Moreover, vehicle lentivirus vectors that did not contain the RNAi sequence were prepared for control. For the rats receiving lentivirus transduction, tooth movement was initiated 7 days after lentivirus transduction.

### Immunostaining

Tissue samples were cryosectioned at a thickness of 10 µm and thaw-mounted on slides coated with poly-L-lysine. Following fixation in cold propanol for 15 min, samples were rinsed with PBS and incubated overnight at 4 °C with primary antibodies against NGF (1:50; Abcam) or ASIC3 (1:100; Alomone), followed by incubation with secondary antibodies at 37 °C for 1 h. Fluorescence microscope (AX10 imager A2/AX10 cam HRC, Zesis) with ZEN Widefield software (Version 2012, Zesis) were used for image acquisition. For quantification of optical density, integrated optical density/area was measured in each of five randomly consecutive fields of the maxillary branch of TG in each of three slides through Image Pro Plus 6.0 (Media Cybernetics, Rockville, MD, USA). Then, the mean value of these 15 fields was regarded as the expression levels of proteins.

### Real-time PCR and western blotting

For real-time PCR, total RNA was extracted from TG by using Takara MiniBEST Universal RNA Extraction Kit (Takara, Shiga, Japan) according to manufacturer’s protocols. Then, cDNA was reversely transcribed by using the M-MLV Kit (Promega), with the heating protocol being initial activation at 95 °C for 3 min, denaturation at 95 °C for 10 s, annealing at 60 °C for 10 s, extension at 72 °C for 20 s and amplification for 40 cycles. Real-time PCR (10 µL) was performed with the aforementioned cDNA (1 µL). The reference gene was β-actin (forward: CACCCGCGAGTACAACCTTC, reverse: CTCAGCACCAGCATCACC). Primers were GCCCAATAAAGGCTTTGCCAAGG (forward) and TGCCTGTACGCCGATCAAAAACG (reverse) for NGF and were CAACCGCAGCGAGTCCTACATTAC (forward) and AGGCTTGCTCCAATAAACAGTCCC (reverse) for ASIC3. For western blotting, tissue samples were homogenized with RIPA lysis buffer. Then, samples were separated by electrophoresis, and proteins were transferred onto polyvinylidene fluoride membranes and incubated with primary antibodies against NGF (1:200; Abcam), ASIC3 (1:200; Alomone), or β-actin (1:10 000; Abcam). Horseradish peroxidase-conjugated secondary antibodies were used for visualization of the proteins. The protein blot densities were analyzed through Image Pro Plus 6.0 (Media Cybernetics, Rockville, MD, USA) with β-actin being the internal reference.

### Statistical analyses

One-way ANOVA with Bonferroni post hoc test and Student’s *t* test were used for statistical analyses. All the statistical analyses were performed in SPSS 16.0 (SPSS, Chicago, USA) and GraphPad Prism 7 (GraphPad Software, San Diego, CA). A *P* value < 0.05 was considered as statistical significance.
